# Whole Genome and Core Genome Multilocus Sequence Typing and Single Nucleotide Polymorphism Analyses of Listeria monocytogenes Isolates Associated with an Outbreak Linked to Cheese, United States, 2013

**DOI:** 10.1128/AEM.00633-17

**Published:** 2017-07-17

**Authors:** Yi Chen, Yan Luo, Heather Carleton, Ruth Timme, David Melka, Tim Muruvanda, Charles Wang, George Kastanis, Lee S. Katz, Lauren Turner, Angela Fritzinger, Terence Moore, Robert Stones, Joseph Blankenship, Monique Salter, Mickey Parish, Thomas S. Hammack, Peter S. Evans, Cheryl L. Tarr, Marc W. Allard, Errol A. Strain, Eric W. Brown

**Affiliations:** a Food and Drug Administration, College Park, Maryland, USA; b Centers for Disease Control and Prevention, Atlanta, Georgia, USA; c Virginia Division of Consolidated Laboratory Services, Richmond, Virginia, USA; d Maryland Department of Health and Mental Hygiene, Baltimore, Maryland, USA; e Newcastle University, Newcastle upon Tyne, United Kingdom; University of Michigan—Ann Arbor

**Keywords:** whole genome multilocus sequence typing, core genome multilocus sequence typing, whole genome sequencing, Listeria monocytogenes, outbreak

## Abstract

Epidemiological findings of a listeriosis outbreak in 2013 implicated Hispanic-style cheese produced by company A, and pulsed-field gel electrophoresis (PFGE) and whole genome sequencing (WGS) were performed on clinical isolates and representative isolates collected from company A cheese and environmental samples during the investigation. The results strengthened the evidence for cheese as the vehicle. Surveillance sampling and WGS 3 months later revealed that the equipment purchased by company B from company A yielded an environmental isolate highly similar to all outbreak isolates. The whole genome and core genome multilocus sequence typing and single nucleotide polymorphism (SNP) analyses results were compared to demonstrate the maximum discriminatory power obtained by using multiple analyses, which were needed to differentiate outbreak-associated isolates from a PFGE-indistinguishable isolate collected in a nonimplicated food source in 2012. This unrelated isolate differed from the outbreak isolates by only 7 to 14 SNPs, and as a result, the minimum spanning tree from the whole genome analyses and certain variant calling approach and phylogenetic algorithm for core genome-based analyses could not provide differentiation between unrelated isolates. Our data also suggest that SNP/allele counts should always be combined with WGS clustering analysis generated by phylogenetically meaningful algorithms on a sufficient number of isolates, and the SNP/allele threshold alone does not provide sufficient evidence to delineate an outbreak. The putative prophages were conserved across all the outbreak isolates. All outbreak isolates belonged to clonal complex 5 and serotype 1/2b and had an identical *inlA* sequence which did not have premature stop codons.

**IMPORTANCE** In this outbreak, multiple analytical approaches were used for maximum discriminatory power. A PFGE-matched, epidemiologically unrelated isolate had high genetic similarity to the outbreak-associated isolates, with as few as 7 SNP differences. Therefore, the SNP/allele threshold should not be used as the only evidence to define the scope of an outbreak. It is critical that the SNP/allele counts be complemented by WGS clustering analysis generated by phylogenetically meaningful algorithms to distinguish outbreak-associated isolates from epidemiologically unrelated isolates. Careful selection of a variant calling approach and phylogenetic algorithm is critical for core-genome-based analyses. The whole-genome-based analyses were able to construct the highly resolved phylogeny needed to support the findings of the outbreak investigation. Ultimately, epidemiologic evidence and multiple WGS analyses should be combined to increase confidence levels during outbreak investigations.

## INTRODUCTION

Listeria monocytogenes can survive and/or reproduce in a wide variety of foods and environmental reservoirs and cause foodborne outbreaks (1). For many years, pulsed-field gel electrophoresis (PFGE) has been the gold standard for laboratory analysis of food and clinical isolates for Listeria outbreak investigations. However, PFGE does not provide a measure of phylogenetic relatedness, and thus, highly related L. monocytogenes isolates may exhibit different PFGE patterns and isolates that are not related might be indistinguishable by PFGE (2). In contrast, whole genome sequencing (WGS) analysis is more phylogenetically relevant, and a variety of WGS tools have been implemented by public health laboratories in different countries to perform real-time or retrospective molecular epidemiological analyses of L. monocytogenes. Some WGS analytical approaches have targeted the entire genome of L. monocytogenes (2–4), while others have targeted the core genome (5–7). The precision of WGS allows different approaches to assess genomic variations: single nucleotide polymorphisms (SNPs) (8, 9), allelic profiles (2, 4–7), and k-mers (10). To support the rapid archiving and dissemination of WGS data related to foodborne illnesses, the United States launched the GenomeTrakr network of state, federal, and international public health laboratories; this network now has participants from around the world sharing genome sequencing data along with relevant metadata (11). PulseNet has also added WGS to its structure and toolbox to facilitate routine application of WGS in public health laboratories (2). The WGS data are housed in the National Center for Biotechnology Information (NCBI) and are used to generate an SNP-based WGS tree with daily updates (https://www.ncbi.nlm.nih.gov/pathogens/isolates/). This tree, which contains over 14,000 L. monocytogenes genomes to date, provides an initial signal of clusters to be followed by additional WGS analyses and epidemiologic investigation. In the past 3 years, the implementation of WGS for global epidemiological surveillance has assisted in the investigations of numerous listeriosis outbreaks, some of which were multinational outbreaks (2, 8, 12).

Between late 2013 and early 2014, a listeriosis outbreak was initially recognized by PFGE and ultimately included 7 Hispanic patients in Maryland and one in California (13). All patient isolates were serotype 1/2b and indistinguishable by PFGE (13). A PFGE-indistinguishable isolate collected from a cheese product in New York in 2012 was then found in the PulseNet database. The PFGE pattern was rare and was seen only among isolates analyzed during the outbreak investigation. Epidemiological investigation, based on interviews of patients, determined that all patients in Maryland available for interview reported consuming Hispanic-style cheese and shopping at different locations of a small grocery chain (13). Subsequent testing of Hispanic-style cheese that were sold by this grocery chain and were produced by company A in Delaware yielded L. monocytogenes. Patients did not report consuming cheese produced by the New York company that made the 2012 cheese product, and the investigation did not identify any connections between the New York company and company A in Delaware (13). No food history for the patient in California was available (13); this patient was at the time considered part of the outbreak because (i) the onset date of illness was within the same time window as the patients in Maryland, (ii) the isolate appeared to be fairly similar to the Maryland isolates by WGS, and (iii) the outbreak PFGE pattern was rare (13). Given the improvements in resolution made possible by genome sequence-based surveillance, WGS was used to determine the genetic relatedness of the isolates under investigation to complement the epidemiologic data. Multiple federal and state agencies performed WGS on representative company A food samples and environmental isolates, the Maryland and California clinical isolates, and the New York cheese isolate.

Three months after that WGS analysis was completed, routine surveillance and real-time WGS of L. monocytogenes-positive samples identified an environmental isolate from company B that matched the outbreak-associated isolates. An internal FDA investigation discovered that company B in Delaware had purchased equipment from company A; no connection between company B and the New York cheese company was identified. We describe here the WGS analyses of the outbreak-associated clinical, food, and environmental L. monocytogenes isolates performed using multiple methods: a whole genome SNP-based approach (14) performed during the outbreak investigation, a core genome SNP-based approach, a whole genome multilocus sequence typing (wgMLST) scheme (2), and a core genome multilocus sequence typing (cgMLST) scheme (5), all performed retrospectively for comparisons.

## RESULTS

### Isolates.

All the isolates recovered from food and environmental samples from company A and company B were serotype 1/2b and exhibited the same PFGE pattern observed in clinical isolates, with PulseNet pattern assignment of GX6A16.0259/GX6A12.2046 (AscI/ApaI). It was a rare combination pattern in the entire PulseNet database, as it was seen only among isolates analyzed in the present study. *In silico* MLST showed all isolates had MLST sequence type 5 (ST5), were part of clonal complex 5 (CC5), alternatively classified as epidemic clone VI (15). The outbreak strain contained internalins A, B, C, E, F, H, J, K, and P and Listeria pathogenicity island 1 (LIPI-1) (16), but it did not contain LIPI-3 (17) or LIPI-4 (5). *inlA* in the outbreak isolates did not have premature stop codons. These features were the same as the CC5 strains associated with a recent outbreak linked to contaminated ice cream (8).

### SNP-based analyses.

**FIG 1:**
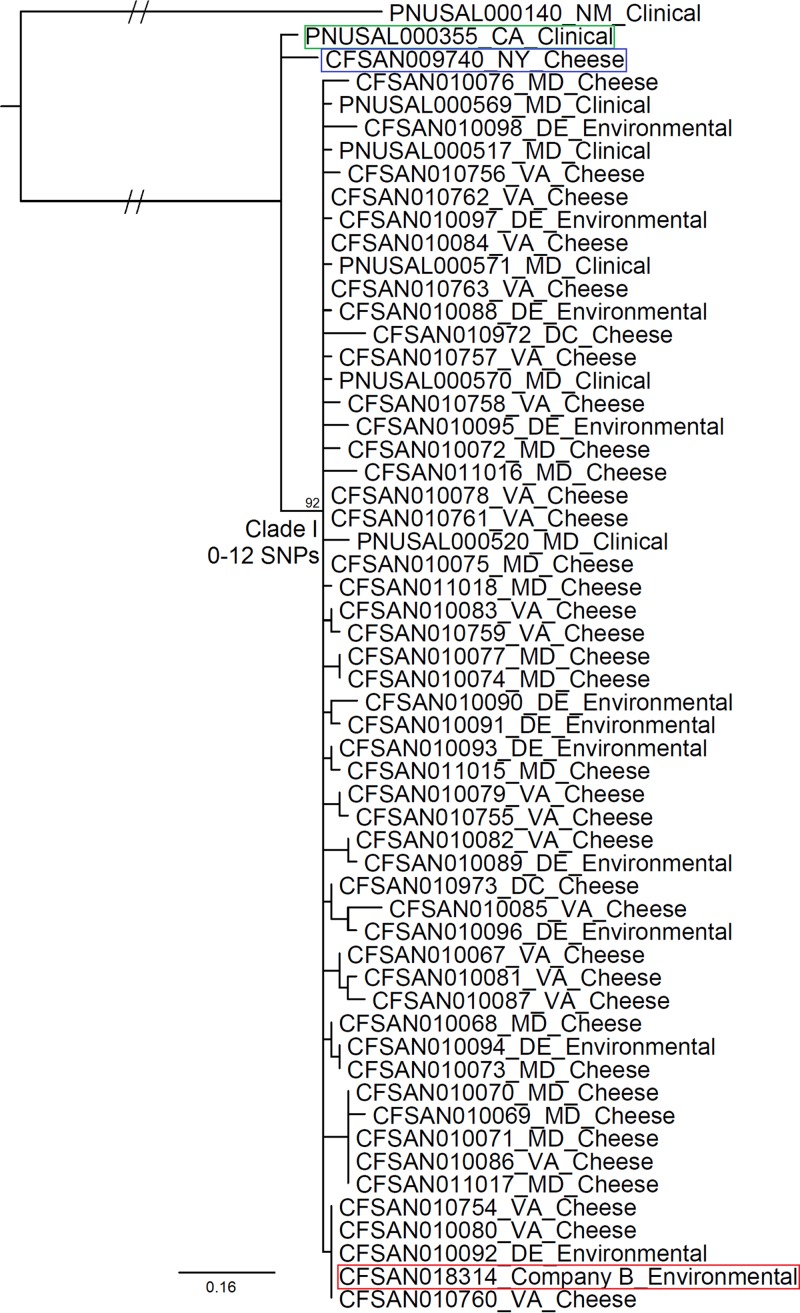
Maximum likelihood tree constructed from SNPs identified by using the CFSAN SNP Pipeline. Isolate identifiers are followed by the abbreviation of the state where they were isolated and the type of sample. The bootstrap value for clade I and the minimum and maximum numbers of pairwise chromosomal SNPs among clade I isolates are listed near the root. The environmental isolate from company B, the New York (NY) cheese isolate, and the California (CA) clinical isolate are highlighted in red, blue, and green boxes, respectively.

Phylogenetic analysis using whole genome SNPs identified by the FDA Center for Food Safety and Applied Nutrition (CFSAN) SNP Pipeline placed the Maryland clinical isolates as well as the company A and company B food and environmental isolates into one clade, clade I. The subclades did not show any association with sample types or sources of sample collection; the 2013 California clinical isolate (PNUSAL000355) and 2012 New York cheese isolate (CFSAN009740) were both placed outside clade I (Fig. 1). A 2013 clinical isolate from New Mexico (PNUSAL000140) of ST5 with a distinct PFGE pattern, which we chose as the outgroup, was clearly distant from all other isolates (Fig. 1), even though it shared the same ST as the outbreak-associated isolates.

The SNP-based WGS analysis showed that the California clinical isolate, the New York cheese isolate, and all clade I isolates except CFSAN010088 had an identical plasmid sequence. CFSAN010088 differed from other isolates by one SNP in the plasmid. Thus, we refer exclusively to SNPs on the chromosome among different isolates in the discussion below. Without counting gaps, clade I isolates differed by 0 to 12 SNPs (median, 4) (Fig. 1). The California clinical isolate differed from clade I isolates by 10 to 17 SNPs (median, 12), and the New York cheese isolate differed from clade I isolates by 7 to 14 SNPs (median, 9). The New Mexico clinical isolate differed from clade I isolates by at least 200 SNPs. The relatively large number of outbreak-associated isolates allowed the identification of specific SNPs that distinguished all clade I isolates from CFSAN009740 (3 nonsynonymous, 3 synonymous, and one noncoding SNP) and all clade I isolates from PNUSAL000355 (4 nonsynonymous, 5 synonymous, and one noncoding SNP) (Table 1). We then chose a subset of the polymorphic loci that were in the cgMLST core coding genome (5), and the maximum likelihood algorithm based on these core coding SNPs placed the California clinical isolate and the New York cheese isolate outside clade I (see Fig. S1 in the supplemental material), congruent with the whole genome SNP analysis. Clade I isolates differed by 0 to 9 SNPs in the core genome. The New York isolate differed from one clade I isolate, CFSAN010085, by 8 core SNPs and differed from other clade I isolates by 3 to 7 core SNPs; the numbers of differences were smaller than the numbers of SNPs between some clade I isolates. The whole genome kSNP analysis also placed these two isolates outside clade I, which contains outbreak-associated isolates (Fig. S2).

**TABLE 1:** Single nucleotide polymorphisms that specifically distinguished clade I isolates from the cheese isolate from New York (CFSAN009740) and clinical isolate from California (PNUSAL000355)^*a*^

SNP position	Nucleotide at the position in isolate(s) from:			Synonymous change?	Amino acid at the position in isolate(s) from:			Gene locus tag, putative protein function, and corresponding gene locus tag in wgMLST pan-genome
	Clade I	NY	CA		Clade I	NY	CA	
479720	T	C^*b*^	C	Yes				CG42_RS02440, ZIP family metal transporter, lmo0414
607603	G	G	T	No	A	A	E	CG42_RS02995, LacI family transcriptional regulator, lmo0535
782555	T	C	C	Yes				CG42_RS03880, flagellar cap protein FliD, lmo0707
1080475	A	G	G	No	E	G	G	CG42_RS05405, copper homeostasis protein CutC, lmo1018
1298795^*c*^	C	C	A	No	P	P	Q	CG42_RS06585, DNA primase, LMON_1266
1334724	C	T	C	No	T	I	T	CG42_RS06775, histidine phosphatase family protein, lmo1244
1740888	T	C	C	Yes				CG42_RS08730, VOC family protein, lmo1635
1762440	C	G	G					Intergenic
1775838	C	A	C	No	A	D	A	CG42_RS08875, rRNA methyltransferase, lmo1662
2275331	T	T	G	Yes				CG42_RS11330, sugar ABC transporter substrate-binding protein, lmo2125
2311944	A	A	G	Yes				CG42_RS11530, xylose isomerase, lmo2160
2532881	C	C	A	No	W	W	L	CG42_RS12665, glutamate decarboxylase, lmo2434

a The reported SNP position, protein ID, and putative functions are based on the complete and annotated chromosome of isolate CFSAN010068 (GenBank accession number NZ_CP014250.1). All specific SNPs are located on the chromosome.

b Underlining indicates that the nucleotide is different from that in clade I isolate.

c The locus is in the putative prophage region.

### wgMLST and cgMLST analyses.

**FIG 2:**
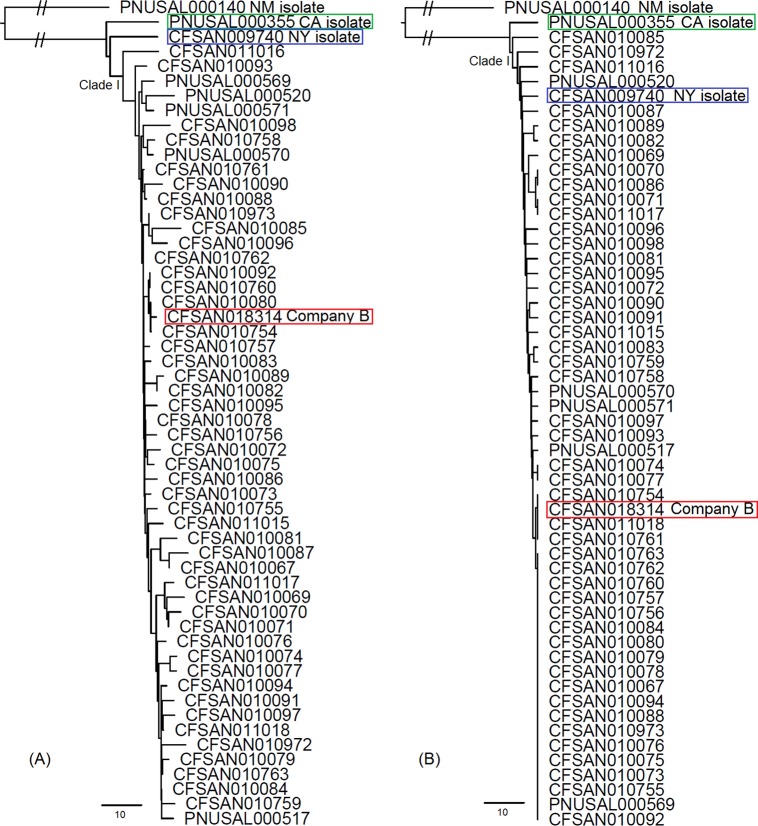
Phylogenetic trees constructed based on wgMLST loci that had summary allele calls for at least one isolate, based on NJ by wgMLST (A) and UPGMA by cgMLST (B). The company B isolate, the New York (NY) cheese isolate, and the California (CA) clinical isolate are highlighted in red, blue, and green boxes, respectively.

**FIG 3:**
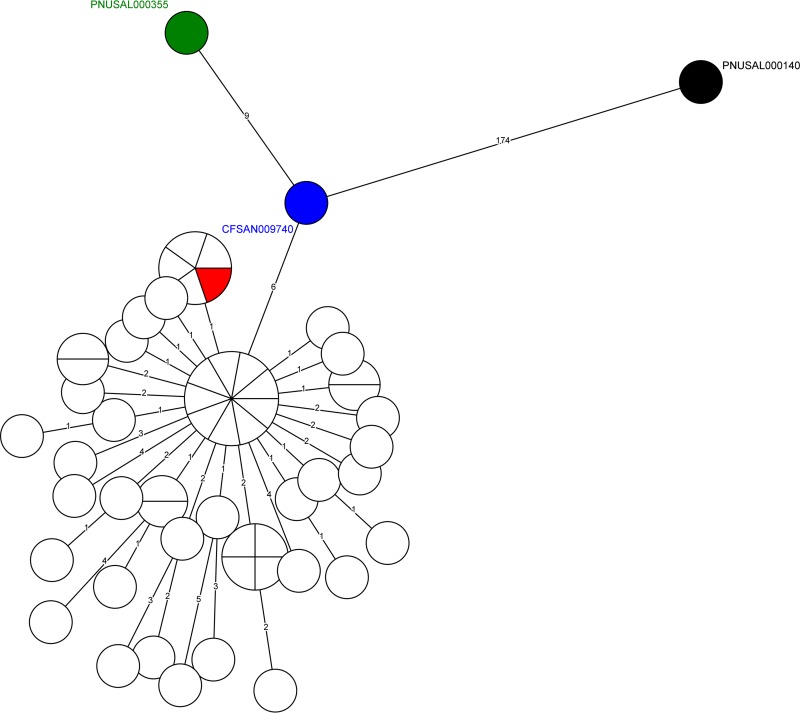
Minimum spanning tree based on wgMLST loci that had summary allele calls for all the isolates. Clade I isolates illustrated in Fig. 1 and 2, except the company B environmental isolate, are shown in white circles, and isolate identifiers are not shown. The New Mexico clinical isolate, California clinical isolate, New York cheese isolate, and company B environmental isolate are in black, green, blue, and red, respectively. The area of each circle is proportional to the number of isolates represented. The number of allele differences between two circles is listed on the line connecting the two circles. The length of each connecting line is proportional to the log of the number of allele differences.

For wgMLST using allele calls combining the assembly-free and assembly-based approaches (designated summary calls via use of BioNumerics 7.5 [Applied Maths, Sint-Martens-Latem, Belgium]), both neighbor-joining (NJ) and unweighted pair group method with arithmetic mean (UPGMA) algorithms generated congruent clustering as the SNP-based analysis did: the New York cheese isolate and the California clinical isolate were placed outside clade I, which contains isolates from food and environmental samples from company A and company B and from patients from Maryland (Fig. 2A; Fig. S3). The New Mexico clinical isolate was distant from all other isolates. The minimum spanning tree (MST) did not clearly illustrate the differentiation between the New York cheese isolate and clade I isolates because they were genetically close (Fig. 3). The alleles were identified that specifically distinguished all clade I isolates from the New York cheese and California clinical isolates (Table 2). The NJ algorithm using cgMLST summary calls generated a clustering congruent with the wgMLST trees, placing the New York cheese and California clinical isolates outside clade I (Fig. S4). Clade I isolates differed from each other by 0 to 9 alleles. The New York isolate differed from a clade I isolate, CFSAN010085, by 8 alleles and differed from other clade I isolates by 3 to 7 alleles, an amount smaller than the maximum pairwise distance among clade I isolates. In contrast, the UPGMA algorithm using cgMLST summary calls placed the New York cheese isolate in clade I (Fig. 2B).

**TABLE 2:** Alleles that specifically distinguished clade I isolates from the cheese isolate from New York (CFSAN009740) and the clinical isolate from California (PNUSAL000355)

Locus in the pan-genome	Allele profile for isolate(s) from:			Putative protein function and corresponding gene locus tag in CFSAN010068 genome
	Clade I	NY	CA	
lmo0414^*a*^	88^*b*^	7^*c*^	7	ZIP family metal transporter,^*d*^ CG42_RS02440
lmo0459^*a*^	5	5	117	Transcriptional regulator, CG42_RS02650
lmo0460^*a*^	14	14	105	Membrane-associated lipoprotein, CG42_RS02660
lmo0535	5	5	115	LacI family transcriptional regulator, CG42_RS02995
lmo0707	100	10	10	Flagellar cap protein FliD, CG42_RS03880
lmo1018	84	108	108	Copper homeostasis protein CutC, CG42_RS05405
LMON_1266^*a*^^,^^*e*^	4	4	24	DNA primase, CG42_RS06585
lmo1244^*a*^	13	128	13	Histidine phosphatase family protein, CG42_RS06775
lmo1337	4	4	117	Rhomboid family intramembrane serine protease,^*d*^ CG42_RS07240
lmo1635^*a*^	36	6	6	VOC family protein,^*d*^ CG42_RS08730
lmo1662	11	134	11	rRNA methyltransferase,^*d*^ CG42_RS08875
lmo2125	2	2	119	Sugar ABC transporter substrate-binding protein, CG42_RS11330
lmo2160^*a*^	17	17	122	Xylose isomerase,^*d*^ CG42_RS11530
lmo2434	15 or 129	15	117	Glutamate decarboxylase, CG42_RS12665

a The locus was included in the wgMLST scheme but not in the cgMLST scheme.

b In the BioNumerics allele database, numbers to designate the same alleles for CDC users are different from those for general users.

c Underlining indicates that the nucleotide is different from that in clade I isolates.

d The functions of genes were identified as hypothetical proteins in the EGD-e annotation (GenBank accession number NC_003210.1), and so the functions of corresponding regions in isolate CFSAN010068 (GenBank accession number NZ_CP014250.1) are listed.

e The locus was identified from the complete genome of EGD (NC_022568.1) as part of the pan-genome panel. The designations for other loci are from the EGD-e genome.

For wgMLST using only assembly-free allele calls or only assembly-based allele calls, both NJ and UPGMA phylogenies placed the New York cheese and California clinical isolates outside clade I, consistent with the phylogeny based on summary calls (Fig. S5, S6, S7, and S8). For cgMLST, the NJ phylogeny using only assembly-based calls was congruent with that using the summary calls, placing the New York cheese and California clinical isolates outside clade I (Fig. S9); however, the NJ phylogeny based on only assembly-free calls placed the New York cheese isolate in clade I (Fig. S10), possibly because in some isolates more loci had no allele calls by assembly-free calling than by assembly-based and summary calling. The UPGMA phylogenies based on only assembly-free calls and only assembly-based calls for cgMLST were congruent with that based on summary calls, placing the New York cheese isolate inside clade I (Fig. S11 and S12).

### Prophage analysis.

The combination of PHAST-based (18) and PHASTER-based (19) analyses of the fully closed genome of CFSAN010068 predicted 2 putative complete prophages, designated prophage 1 (position 68,171 to 115,163) and prophage 2 (position 1,281,529 to 1,324,833). BLAST analyses showed that the 2 putative regions were conserved, with ≥99% query coverage (percentage of the query sequence that overlapped the subject sequence) and ≥99% sequence identity among PFGE-matched isolates: clade I isolates, the New York cheese isolate, and the California clinical isolate. BLAST analyses further showed that prophage 1 was absent (BLAST query coverage of 18%) in the New Mexico clinical isolate (PNUSAL000140), which exhibited a different PFGE pattern, and that the alignment of prophage 2 between the New Mexico clinical isolate and CFSAN010068 had 80% BLAST query coverage, indicating more diversity than that in prophage 2 among PFGE-matched isolates.

## DISCUSSION

### These data confirm that WGS is a useful tool for laboratory analysis during investigations of listeriosis outbreaks.

To integrate the enhanced information of WGS analyses into public health investigations, the FDA and CDC established a real-time Listeria project (2, 11), in which virtually all clinical isolates and the majority of food and environmental isolates of L. monocytogenes collected in the United States are now sequenced and archived, and those genomic data are publicly available. This is a case in which real-time WGS was used by multiple federal and state agencies during the laboratory analysis of food and environmental isolates to support findings of an epidemiological investigation of a listeriosis outbreak in the United States. Data from the real-time Listeria project led to the identification of the transmission of the outbreak strain from company A to company B. The WGS analyses clustered all Maryland clinical isolates with company A food and environmental isolates collected as part of the outbreak investigation and implicated by the epidemiological investigation, and the analysis also excluded the PFGE-indistinguishable isolate collected from an epidemiologically unrelated food source in New York. Although the isolate from the California patient was relatively closely related to the outbreak-associated isolates, further WGS analyses performed after the investigation's conclusion did not support the inclusion of this patient's illness as part of the outbreak. The food history for this patient was not available to allow suggestions of any alternative food sources for sampling and testing (13). Nonetheless, WGS analyses corroborated the conclusion based on the epidemiological investigation on food histories of Maryland patients: company A cheese products were the likely source of the outbreak. The WGS clustering and the small number of SNPs/alleles differentiating clade I isolates from the California clinical isolate and the New York cheese isolate indicated that all of these isolates descended from a very recent common ancestor, which we hypothesize existed outside company A.

### WGS data allowed a side-by-side comparison of WGS analysis methods.

MLST-based methods only consider variants in coding regions. In addition, they count all variants in one coding region as one allele difference, to correct for recombination events that account for multiple variants in one region (33). Thus, MLST methods inherently offer less resolution than whole-genome-wide variants. However, the performance of a specific MLST method or a specific SNP-based method is also affected by the allele/SNP calling algorithms. For example, an indel results in a different allele call by wgMLST, but it would not be counted by the CFSAN SNP Pipeline unless at least one other isolate had an SNP in that nucleotide position. The CFSAN SNP Pipeline employs a filter to remove SNPs that may be the result of recombination or low-quality sequencing/mapping. BioNumerics also employs algorithms to process questionable wgMLST calls (discussed below). In this study, we used an outbreak-associated isolate (CFSAN010068) as the reference for the CFSAN SNP Pipeline to increase the mapping quality (20). This genome was completely closed to maximize the resolution of variant calling. wgMLST identified 6 alleles that specifically distinguished the entire clade I from the New York cheese isolate, and 3 of the alleles were not targeted by cgMLST (Table 2). wgMLST also identified 2 other alleles (lmo2691 and lmo2434) in which the New York cheese isolate differed from at least 5 of the clade I isolates; lmo2691 was not targeted by cgMLST. This may explain why the UPGMA algorithm or assembly-free allele calling by cgMLST placed the New York cheese isolate into clade I. When we compared the New York cheese isolate with clade I isolates, the specific SNPs identified by the CFSAN SNP Pipeline were concordant with the specific alleles identified by wgMLST (Tables 1 and 2). However, when comparing the California clinical isolate with clade I isolates, there were differences between the CFSAN SNP Pipeline and wgMLST. Specifically, the SNP Pipeline identified an SNP in an intergenic region, which was not targeted by wgMLST. In the reference genome (CFSAN010068) regions corresponding to the 3 wgMLST allele mismatches (lmo1337, lmo0459, and lmo0460), the SNP Pipeline did not call any SNPs (Tables 1 and 2). We then checked the raw reads mapping and found a single nucleotide deletion in the genomic region corresponding to lmo1337 in the California clinical isolate. The indel in this isolate resulted in a different allele call by wgMLST, but it was not counted by the SNP Pipeline because no other isolates had an SNP in the same nucleotide position. Examination of raw reads confirmed DNA variations in the genomic regions corresponding to lmo0459 and lmo0460 in the California clinical isolate. Through the use of the Tandem Repeats Finder program (21), we discovered that those variations were in a tandem repeat region (data not shown), which would be challenging to resolve by next-generation sequencing and often generates false high-density SNPs with reads mapping (8, 22). This explains why they were filtered from the final SNP matrix by the SNP Pipeline. Thus, the use of multiple WGS analysis approaches maximized the discovery of genetic variants, which illustrates that using multiple tools could help exclude unrelated isolates in future investigations where isolates are highly similar to each other.

The BioNumerics process, used at the default setting, combines the call generated by the assembly-based approach and the call generated by the assembly-free approach into a summary call for each locus. Briefly, when the two approaches yield an identical call, that call is the summary call; when the two approaches yield different calls, there is be no summary allele call; when one approach yields an allele call and the other approach yields no allele call, the summary call is the call yielded by the first approach. NJ and UPGMA are two common phylogenetic algorithms for analyzing allele profiles. In this study, the summary calls, assembly-based calls, and assembly-free calls yielded the same NJ and UPGMA wgMLST phylogenies between the New York/California isolates and clade I isolates, despite minor differences in the subclades within clade I, which were expected. However, the assembly-free calls for cgMLST could not differentiate the New York isolate from clade I isolates. In the future, even for the same wgMLST/cgMLST target gene set, different software to implement the analyses, different allele-calling algorithms, or different parameters of the same allele-calling algorithm should be thoroughly evaluated using more outbreak data sets, especially when epidemiologically unrelated isolates exhibit high genetic similarity to the outbreak isolates.

The kSNP analysis corroborated the results of the SNP analysis method and of wgMLST in the identification of the food source of this outbreak. However, the utility of the kSNP approach for routine identification of outbreak clusters still needs further evaluation (23).

### WGS phylogeny is critical for identifying the scope of an outbreak and we cannot solely rely on the SNP/allele threshold.

The accuracy of SNP calling in reference-based methods can be reduced when they are applied to relatively diverse genomes; that is why when the determination of the number of SNPs among isolates is critical, it is preferable to remove the relatively distant outgroup for a second analysis (8). In this data set, removing the New Mexico isolate from the SNP Pipeline analysis did not change the SNP calling among other isolates. The same SNP-based analysis and wgMLST analysis revealed that isolates involved in other outbreaks had various degree of diversity, with 4 to 42 SNPs (8, 24, 25) or 5 to 43 alleles (2, 12, 26, 27). Isolates could accumulate various degrees of genetic variations after entering a food-processing facility; alternatively, isolates from a common source could evolve for years, accumulating genetic variations, prior to entering a facility through a single or multiple contamination events. In some other WGS studies of listeriosis outbreaks, the minimum number of SNP/allele differences between outbreak-associated isolates and epidemiologically unrelated isolates was more than 3 times the maximum number of pairwise SNP/allele differences among outbreak-associated isolates (9, 28). These studies either did not employ any molecular subtyping tools or employed MLST for screening suspect/background isolates (9, 28). In contrast, we used PFGE, which has greater discriminatory power than MLST, to screen for suspect/background isolates more likely to be genetically close to the outbreak isolates, and we included them in the epidemiologic investigation and WGS analysis. This approach was also used in some other studies (4, 29). Our data showed that the numbers of SNP/allele differences between clade I isolates and the epidemiologically unrelated, PFGE-matched New York and California isolates were not much larger than those among clade I isolates. In contrast, the MLST-matched New Mexico clinical isolate, which had a different PFGE profile from clade I isolates, differed from clade I isolates by more than 200 SNPs. The high genetic similarity among the PFGE-matched isolates is what led us to conclude that all of these isolates share a recent common ancestor. Since the 2012 New York cheese isolate was known not to be linked to the outbreak, we also believe that the California clinical isolate might not be part of the outbreak. Our exclusion of the New York isolate and the California isolate is not based on an SNP/allele threshold, rather, it is supported by topologies generated using the whole genome SNP matrix, core genome SNP matrix, whole genome k-mer SNP, wgMLST, and NJ topologies generated using cgMLST summary allele calls or assembly-based allele calls. These results highlight why it is critical that genetic differences be complemented by WGS trees generated by phylogenetically meaningful algorithms to distinguish outbreak-associated isolates from epidemiologically unrelated isolates. The sufficient number of food and environmental isolates was also important in generating the meaningful WGS phylogeny.

MLST approaches convert sequence variations to allelic profiles and use distance-based methods to reconstruct phylogeny. NJ and UPGMA are two of the most commonly used distance-based phylogenetic reconstruction algorithms; they build phylogenetic clustering by searching the genetic distance matrix for the most closely related isolates and then connecting these isolates at a node (30). UPGMA assumes a perfect molecular clock and an equal evolutionary rate for all isolates, which is a very rare condition. In contrast, NJ is more flexible as it allows the evolutionary rates to vary by isolates (30). Therefore, NJ incorporates more parameters of evolution for phylogenetic reconstruction and is generally more reliable than UPGMA (30). The purpose of WGS for source tracking is to differentiate outbreak-associated isolates from epidemiologically unrelated isolates. Therefore, if these isolates are genetically distant, the choice of phylogenetic reconstruction algorithms may not be critical. In this outbreak, the number of SNPs/alleles between epidemiologically unrelated isolates was relatively small, but UPGMA topologies based on wgMLST alleles still differentiated epidemiologically unrelated isolates. The number of SNPs/alleles between epidemiologically unrelated isolates in the core genome was even smaller, which exposed the weakness of UPGMA. MSTs display the number of allelic differences between isolates and thus should be interpreted with caution when outbreak-associated isolates are genetically close to epidemiologically unrelated isolates, especially considering that central allelic profiles of MSTs may not be ancestral founders but rather frequent allelic profiles among a group of isolates (31). cgMLST had limited discriminatory power in this investigation, and therefore we suggest that different phylogenetic algorithms be explored with cgMLST and that any cgMLST clustering should be followed by wgMLST and/or whole-genome SNP analysis when using epidemiologic evidence.

### Putative prophages had significant divergence among the MLST-matched isolates but were conserved among the PFGE-matched isolates and not sufficient to exclude epidemiologically unrelated isolates from the outbreak.

There were no major prophage variations among clade I isolates, the California clinical isolate, and the New York cheese isolate; this was consistent with the finding that only one of the SNPs that specifically differentiated PNUSAL000355 from clade I isolates was a putative prophage (Table 1). Thus, the prophage variations did not contribute to the differentiation between the New York/California isolates and clade I isolates. In contrast, the CC5 isolate from New Mexico, which exhibited a different PFGE pattern, significantly differed in prophage profile from those of isolates exhibiting the outbreak PFGE pattern. Thus, PFGE and prophage variations possessed similar discriminatory power, which was lower than that of WGS analysis. The prophage variations resulted in a high density of SNPs between the New Mexico isolate and other isolates and were excluded by the SNP Pipeline because these variations could be the result of recombination and the number of SNPs does not necessarily reflect the evolutionary relatedness among isolates. In some other studies, prophage variations have been more discriminatory than PFGE. For example, PFGE-indistinguishable ST11 isolates that persisted in the same food-processing facility had significant *comK* prophage divergence (32). Prophages were conserved among prophage-containing isolates associated with an ice cream outbreak and were diverse between outbreak isolates and nonoutbreak isolates that were matched by PFGE (8). DNA sequence variations are more informative than PFGE banding patterns, and thus, even in situations in which prophage variations offer similar discriminatory power as PFGE, they are still valuable for studying strain relationships. Some of the insertions/deletions in prophages could be sequencing artifacts; however, we fully closed the reference genome, and the alignment of prophages (>99% coverage) between the closed genome and draft genomes of PFGE-matched isolates indicated that the draft sequencing in this study resolved prophages very well.

### CC5 isolates are involved in more than half of reported invasive listeriosis outbreaks caused by confirmed serotype 1/2b strains.

There are 13 serotypes of L. monocytogenes, with serotypes 4b, 1/2b, and 1/2a associated with the majority of the listeriosis outbreaks (7). The clonal complexes of L. monocytogenes were defined based on allele differences identified by a 7-gene MLST scheme (33), and it was recently demonstrated that the clonal complex definition is generally compatible with WGS clustering (5, 7). Historically, confirmed serotype 1/2b strains (i.e., serotypes confirmed by antisera agglutination, not just by PCR serogrouping) have been mostly associated with gastrointestinal outbreaks, linked to contaminated chocolate milk in Illinois in 1994 (34), contaminated rice salad in Italy in 1993 (35), and contaminated imitation crab salad in Canada in 1996 (36). One patient involved in the Canada crab salad outbreak had an invasive infection, but the symptoms were predominantly gastrointestinal (36). A 1987 Pennsylvania outbreak linked to contaminated salami or ice cream involved serotype 1/2b isolates (CC3) and invasive listeriosis (37). Recently, more invasive listeriosis outbreaks involving serotype 1/2b were reported, including the outbreak we evaluated here (CC5), a cluster of illnesses in the 2011 U.S. cantaloupe outbreak (CC5) (38), a 2013-2014 Spain foie gras outbreak (CC87) (39), a 2013-2014 Spain outbreak with an unidentified food source (CC87) (39), a cluster of illnesses in the 2010-2015 U.S. ice cream outbreak (CC5) (8), and a 2011-2013 Austria outbreak linked to contaminated cheese or meat (CC5) (4). Interestingly, 4 of these 6 outbreaks involved CC5, which indicates this clonal group might have hypervirulent phenotypes or phenotypes that allow more successful persistence in foods and food-processing environments than other serotype 1/2b strains.

### Conclusions.

WGS analysis was a highly useful addition to epidemiologic and trace-back data in the investigation of this outbreak and in tracing the spread of outbreak isolates across more than one food-processing facility. Notably, WGS distinguished outbreak-associated isolates from the PFGE-matched New York cheese isolate collected from an epidemiologically unrelated food source. Additional phylogenetic analysis conducted after conclusion of the outbreak suggested that the California clinical isolate with high genetic similarity to the outbreak isolates was likely not part of the outbreak. The detailed scrutiny of this data set demonstrated that prophage variations, the UPGMA algorithm, or assembly-free allele calling for cgMLST were insufficient for exclusion of the New York cheese isolate that was not associated with the outbreak. From the analyses based on whole genome variations, we were able to construct the highly resolved phylogeny needed for investigation; we should not rely solely on an SNP/allele threshold to delineate an outbreak. Ultimately, a combination of epidemiologic evidence, PFGE data, and multiple WGS analyses should be applied to increase confidence during outbreak investigations.

## MATERIALS AND METHODS

### Isolates.

The following isolates were included in the study: 5 isolates from patients in Maryland obtained in 2013, 1 isolate from the patient in California obtained in 2013, 1 isolate from the cheese sample collected in New York in 2012, and 48 isolates from L. monocytogenes-positive cheese samples of different batches and environmental samples from different company A facility areas collected in 2014; also included was 1 isolate from the company B environmental sample obtained during a regular surveillance sampling 3 months after the outbreak investigation (Table 3). Four of the seven Maryland patients were mother-newborn pairs, for which only the newborn clinical isolates were analyzed. We used the genome sequence of the clinical isolate in New Mexico in 2013 (PNUSAL000140) as the outgroup for the above-mentioned isolates; PNUSAL000140 has the same MLST-based ST as the outbreak-associated isolates, but it has a distinct PFGE pattern.

**TABLE 3:** Isolates analyzed in the present study

Strain identifier	GenBank accession no.	Source state	Sample type	Collection date
PNUSAL000140^*a*^	SRR974871	New Mexico	Clinical	July 2013
PNUSAL000355	SRR1027093	California	Clinical	October 2013
CFSAN009740	SRR1200763	New York	Cheese	December 2012
PNUSAL000569	SRR1174760	Maryland	Clinical	August 2013
PNUSAL000571	SRR1193826	Maryland	Clinical	August 2013
PNUSAL000570	SRR1193825	Maryland	Clinical	August 2013
PNUSAL000517	SRR1112195	Maryland	Clinical	October 2013
PNUSAL000520	SRR1112204	Maryland	Clinical	November 2013
CFSAN011016	SRR1378358	Maryland	Cheese	February 2014
CFSAN011017	SRR1378351	Maryland	Cheese	February 2014
CFSAN011018	SRR1378353	Maryland	Cheese	February 2014
CFSAN010068	NZ_CP014250.1^*b*^	Maryland	Cheese	February 2014
CFSAN010069	SRR1181541	Maryland	Cheese	February 2014
CFSAN010070	SRR1181568	Maryland	Cheese	February 2014
CFSAN010071	SRR1181535	Maryland	Cheese	February 2014
CFSAN010072	SRR1181561	Maryland	Cheese	February 2014
CFSAN010073	SRR1181538	Maryland	Cheese	February 2014
CFSAN010074	SRR1181554	Maryland	Cheese	February 2014
CFSAN010075	SRR1181556	Maryland	Cheese	February 2014
CFSAN010076	SRR1181567	Maryland	Cheese	February 2014
CFSAN010077	SRR1181511	Maryland	Cheese	February 2014
CFSAN011015	SRR1378347	Maryland	Cheese	February 2014
CFSAN010972	SRR1198952	Washington, DC	Cheese	February 2014
CFSAN010973	SRR1198878	Washington, DC	Cheese	February 2014
CFSAN010088	SRR1195636	Delaware	Environment	February 2014
CFSAN010089	SRR1195637	Delaware	Environment	February 2014
CFSAN010090	SRR1195675	Delaware	Environment	February 2014
CFSAN010091	SRR1195661	Delaware	Environment	February 2014
CFSAN010092	SRR1195691	Delaware	Environment	February 2014
CFSAN010093	SRR1186333	Delaware	Environment	February 2014
CFSAN010094	SRR1195629	Delaware	Environment	February 2014
CFSAN010095	SRR1195657	Delaware	Environment	February 2014
CFSAN010096	SRR1195670	Delaware	Environment	February 2014
CFSAN010097	SRR1186346	Delaware	Environment	February 2014
CFSAN010098	SRR1186334	Delaware	Environment	February 2014
CFSAN018314	SRR1555351	Delaware	Environment	May 2014
CFSAN010067	SRR1177313	Virginia	Cheese	February 2014
CFSAN010078	SRR1181539	Virginia	Cheese	February 2014
CFSAN010079	SRR1182716	Virginia	Cheese	February 2014
CFSAN010080	SRR1182219	Virginia	Cheese	February 2014
CFSAN010081	SRR1182220	Virginia	Cheese	February 2014
CFSAN010082	SRR1182225	Virginia	Cheese	February 2014
CFSAN010083	SRR1182221	Virginia	Cheese	February 2014
CFSAN010084	SRR1182222	Virginia	Cheese	February 2014
CFSAN010085	SRR1182223	Virginia	Cheese	February 2014
CFSAN010086	SRR1182224	Virginia	Cheese	February 2014
CFSAN010087	SRR1181522	Virginia	Cheese	February 2014
CFSAN010754	SRR1187613	Virginia	Cheese	February 2014
CFSAN010755	SRR1187589	Virginia	Cheese	February 2014
CFSAN010756	SRR1187587	Virginia	Cheese	February 2014
CFSAN010757	SRR1187440	Virginia	Cheese	February 2014
CFSAN010758	SRR1187427	Virginia	Cheese	February 2014
CFSAN010759	SRR1187445	Virginia	Cheese	February 2014
CFSAN010760	SRR1187584	Virginia	Cheese	February 2014
CFSAN010761	SRR1187420	Virginia	Cheese	February 2014
CFSAN010762	SRR1187616	Virginia	Cheese	February 2014
CFSAN010763	SRR1187425	Virginia	Cheese	February 2014

a All isolates were serotype 1/2b, CC5. All isolates except PNUSAL000140 had the PFGE pattern GX6A16.0259/GX6A12.2046 (AscI/ApaI).

b For identification of SNPs via the CFSAN SNP Pipeline, the completely closed genome of the reference isolate and raw reads from other isolates were used. The closed genome was not used in the wgMLST/cgMLST analyses.

### PFGE and whole genome sequencing.

The standard PulseNet protocol with restriction endonuclease digestion by AscI/ApaI (40) was used to perform the PFGE. One outbreak isolate, CFSAN010068, taken from a company A cheese sample, was selected to be fully sequenced using the PacBio RS II system (Pacific Biosciences, Menlo Park, CA, USA) and achieved at least 100× average genome coverage, as previously described (24, 41). This fully closed genome was used as the reference genome for mapping and SNP calls, as described below. Other isolates were sequenced using the MiSeq V2 kit (Illumina, Inc., San Diego, CA) (two 250-bp-length runs) as previously described (24). All of the sequences were deposited in the FDA GenomeTrakr database (http://www.ncbi.nlm.nih.gov/bioproject/183844) (Table 3).

### SNP analyses.

SNPs were identified using the FDA CFSAN SNP Pipeline v0.6.0 with default settings (3, 14). Briefly, raw reads from each genome were mapped to CFSAN010068 by using Bowtie 2 version 2.2.2 (42). The BAM file was sorted using Samtools version 0.1.19 (43), and a pileup file for each genome was produced. These files were then processed using VarScan2 version 2.3.9 to identify high-quality variant sites (44). The Python script was used to parse the .vcf files and construct an initial SNP matrix. For this set of relatively closely related isolates, the SNP Pipeline applied a filter to exclude variant sites in high-density variant regions (≥3 variant sites in ≤1,000 bp of any one genome), since they may be the result of recombination or low-quality sequencing/mapping, which often occurred in repetitive regions. The excluded regions combined were 2,632 bp (containing 31 variant sites), 38,051 bp (778 variant sites), and 3,906 bp (31 variant sites). The first two regions were in prophages, containing SNPs only between PNUSAL000140 and other isolates; the third region was a repetitive region containing SNPs only between PNUSAL000355 and other isolates. No excluded regions contained SNPs among other isolates. Detailed information (e.g., code and instructions) is available at https://github.com/CFSAN-Biostatistics/snp-pipeline. GARLI (45) was subsequently used to infer two phylogenies, one based on the SNP matrix in the entire genome and the other based on the SNPs only in the core genome (1,748 coding sequences, as discussed below). A separate k-mer-based approach was also used to generate a whole genome tree, by using kSNP v3 software (46) in order to determine whether the different SNP-based approaches generated concordant clustering.

### wgMLST and cgMLST analyses.

wgMLST and cgMLST analyses were performed using tools in BioNumerics 7.5. Briefly, alleles were identified by the combination of an assembly-free k-mer-based approach using raw reads and assembly-based BLAST approach using SPAdes version 3.5.0-assembled genomes (47) with the wgMLST and cgMLST L. monocytogenes tools within BioNumerics 7.5. The wgMLST scheme contains 4,797 coding loci, representing a pan-genome of L. monocytogenes identified from over 150 previously published genomes (48). Among them, 1,748 coding loci represent the core genome of L. monocytogenes (5). Once all alleles were assigned to each genome, NJ and UPGMA trees were constructed for wgMLST and cgMLST. Loci with no allele calls were ignored in the pairwise comparison during the tree construction. For wgMLST, a subset of loci in which all isolates had allele calls was used to construct an MST based on the allelic profile of each individual isolate. We also performed the same wgMLST/cgMLST analyses using the assembly-free-only approach and assembly-based-only approach in BioNumerics 7.5.

### *In silico* MLST, prophage, and virulence profile analyses.

*In silico* MLST analysis was performed using the tools in BioNumerics 7.5. The presence of major internalins and Listeria pathogenicity islands (5) in these isolates were determined using the tools in BioNumerics 7.5. A combination of PHAST (18) and PHASTER (19) was used to identify putative prophages from the complete genome of CFSAN010068. Sequences of the putative prophages of CFSAN010068 were analyzed via BLAST (49) against SPAdes v3.5.0-assembled draft genomes (47), and the query coverage (percentage of the query sequence that overlaps the subject sequence) and sequence identify of the BLAST alignment were determined.

### Accession number(s).

The WGS sequences were deposited with GenBank under the accession numbers provided in Table 3 (for the complete genome) and were also assigned Sequence Read Archive (SRA) identifiers for draft genomes.

## Supplementary Material

Supplemental material
