# Supplementary material for: Whole Genome and Core Genome Multilocus Sequence Typing and Single Nucleotide Polymorphism Analyses of Listeria monocytogenes Isolates Associated with an Outbreak Linked to Cheese, United States, 2013
Source: Appl Environ Microbiol. 2017 Jul 17;83(15):e00633-17. doi: 10.1128/AEM.00633-17 (PMC5514676; doi:10.1128/AEM.00633-17)
Supplement: Supplemental material [file supp_83_15_e00633-17__index.html]

Whole Genome and Core Genome Multilocus Sequence Typing and Single Nucleotide Polymorphism Analyses of Listeria monocytogenes Isolates Associated with an Outbreak Linked to Cheese, United States, 2013 — Supplemental material 

# Whole Genome and Core Genome Multilocus Sequence Typing and Single Nucleotide Polymorphism Analyses of Listeria monocytogenes Isolates Associated with an Outbreak Linked to Cheese, United States, 2013

## Supplemental material

- Supplemental file 1 -

  Maximum-likelihood phylogeny based on core genome SNPs (Fig. S1); phylogeny constructed using kSNP v3 (Fig. S2); UPGMA phylogeny based on summary calls of wgMLST (Fig. S3); NJ phylogeny based on summary calls of cgMLST (Fig. S4); NJ phylogeny generated using assembly-based alleles of wgMLST (Fig. S5); UPGMA phylogeny generated using assembly-based alleles of wgMLST (Fig. S6); NJ phylogeny generated using assembly-free alleles of wgMLST (Fig. S7); UPGMA phylogeny generated using assembly-free alleles of wgMLST (Fig. S8); NJ phylogeny generated using assembly-based allele calls of cgMLST (Fig. S9); NJ phylogeny generated using assembly-free allele calls of cgMLST (Fig. S10); UPGMA phylogeny generated using assembly-based allele calls of cgMLST (Fig. S11); UPGMA phylogeny generated using assembly-free allele calls of cgMLST (Fig. S12).

  PDF, 320K
