# Supplementary material for: Whole Genome and Core Genome Multilocus Sequence Typing and Single Nucleotide Polymorphism Analyses of Listeria monocytogenes Isolates Associated with an Outbreak Linked to Cheese, United States, 2013
Source: Appl Environ Microbiol. 2017 Jul 17;83(15):e00633-17. doi: 10.1128/AEM.00633-17 (PMC5514676; doi:10.1128/AEM.00633-17)
Supplement: Supplemental material [file AEM.00633-17_zam999117966s1.pdf]

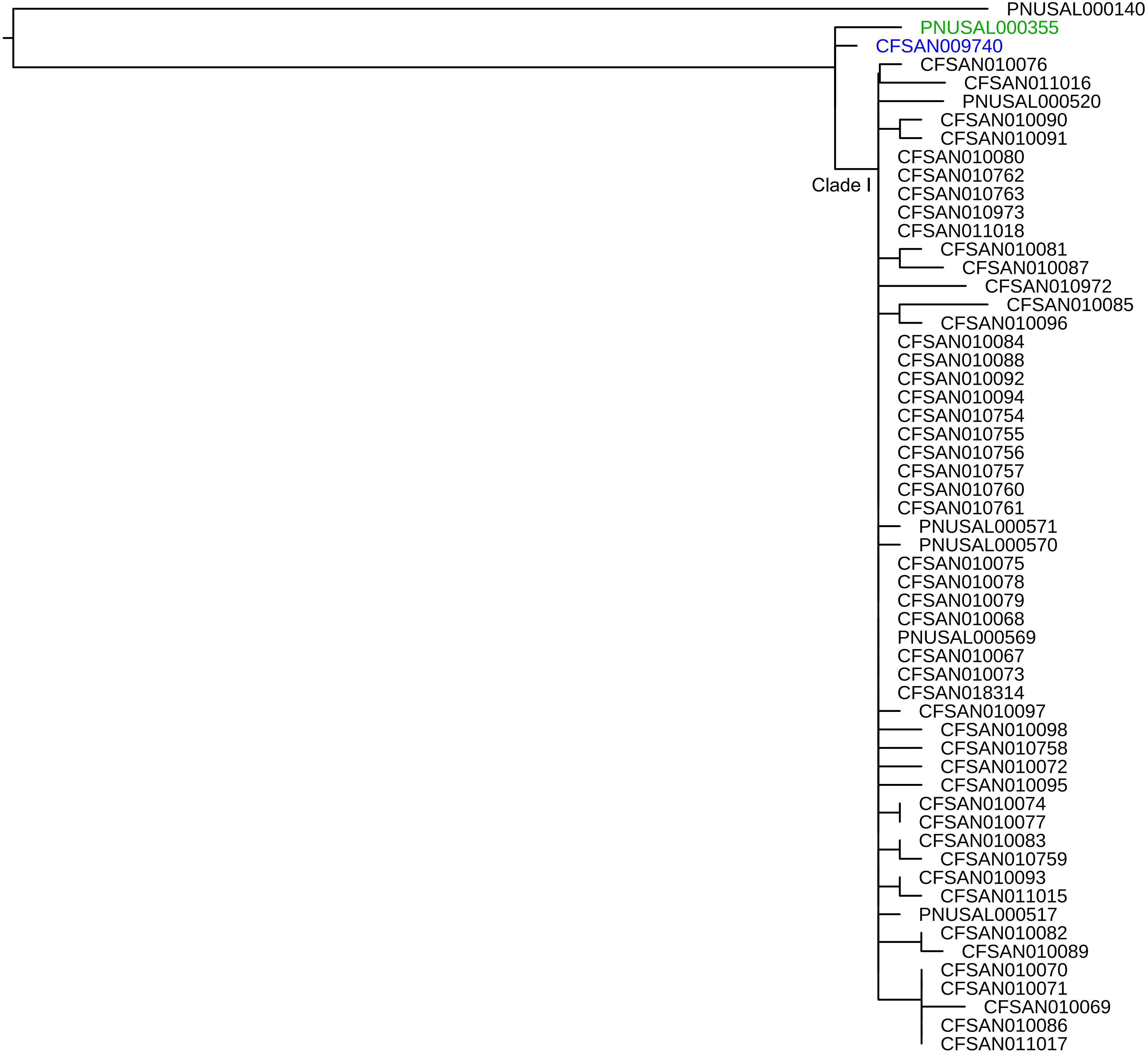

Figure S1. Maximum likelihood phylogeny based on core genome SNPs. The California and New York isolates are marked in green and blue color, respectively.

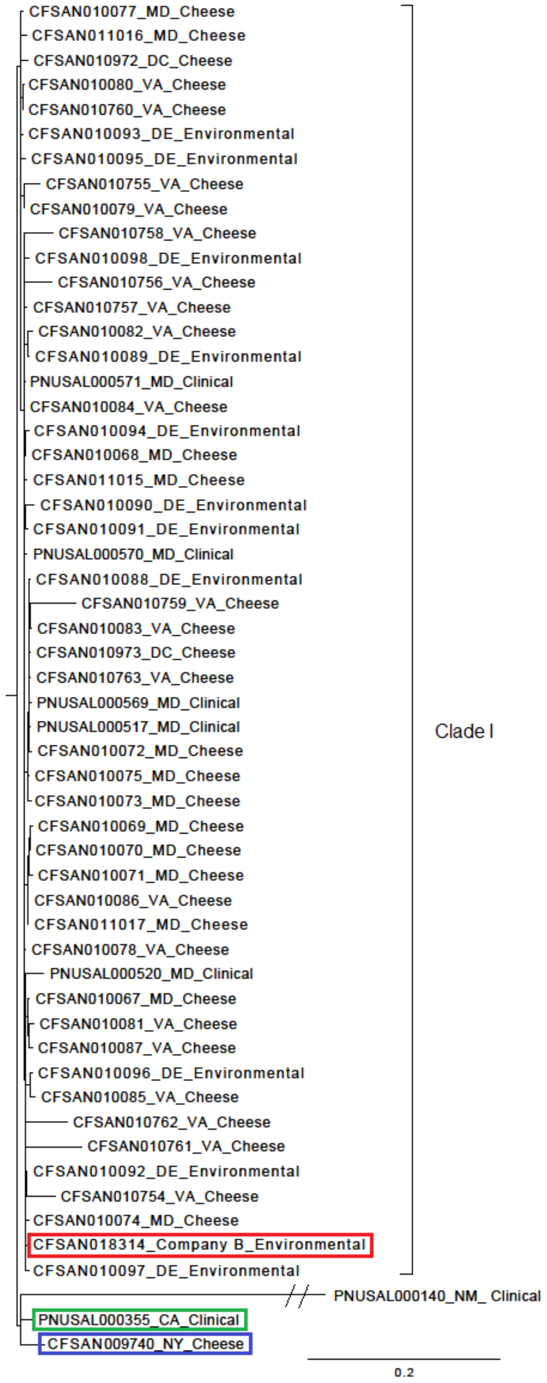

Figure S2. Phylogeny constructed using kSNP v3. Isolate identifiers are followed by the abbreviation of the state where they were isolated and type of samples. The Company B isolate, New York (NY) cheese isolate and California (CA) clinical isolate are highlighted in a red, blue and green box, respectively.

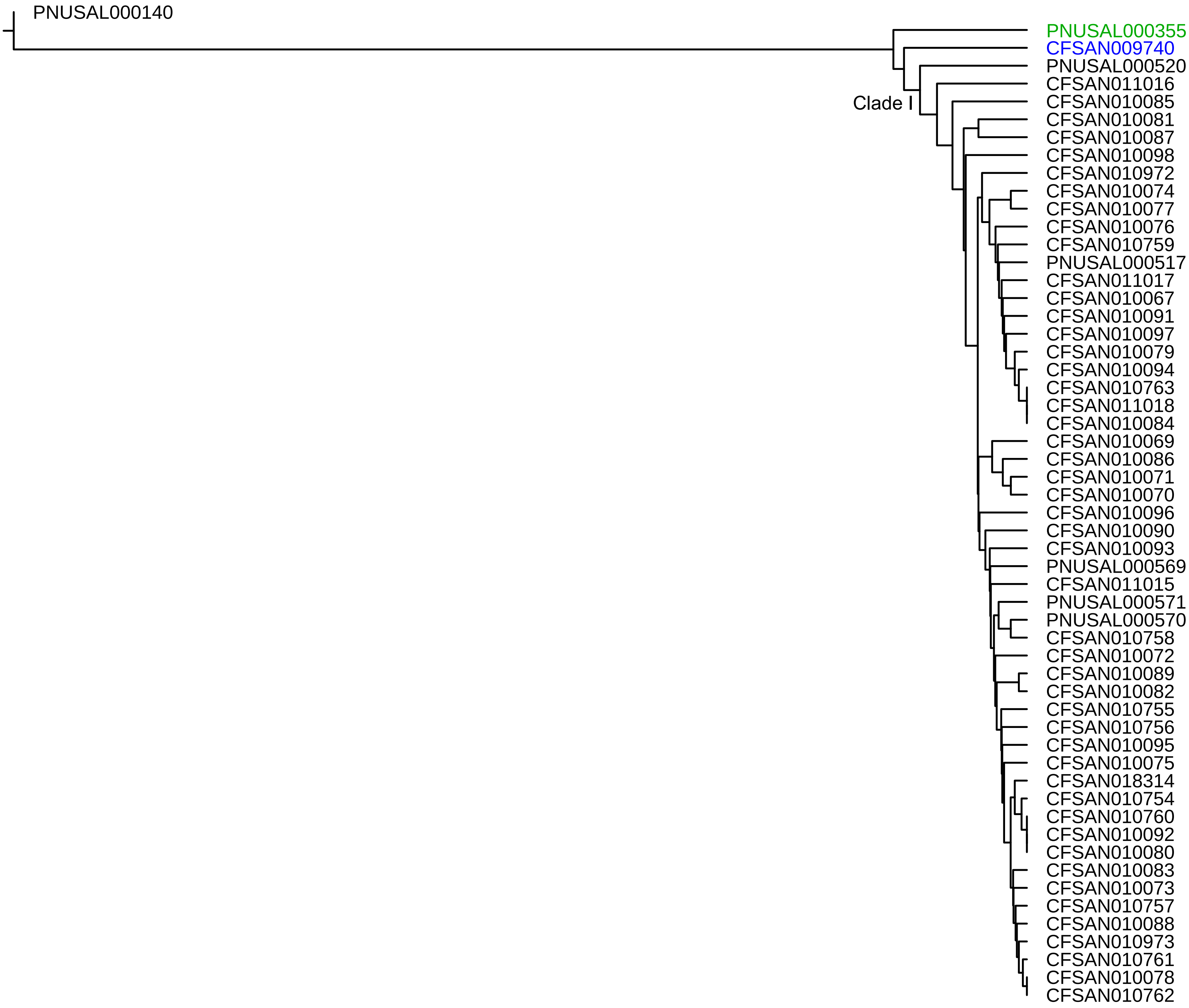

Figure S3. UPGMA phylogeny based on summary calls of wgMLST. The California and New York isolates are marked in green and blue color, respectively.

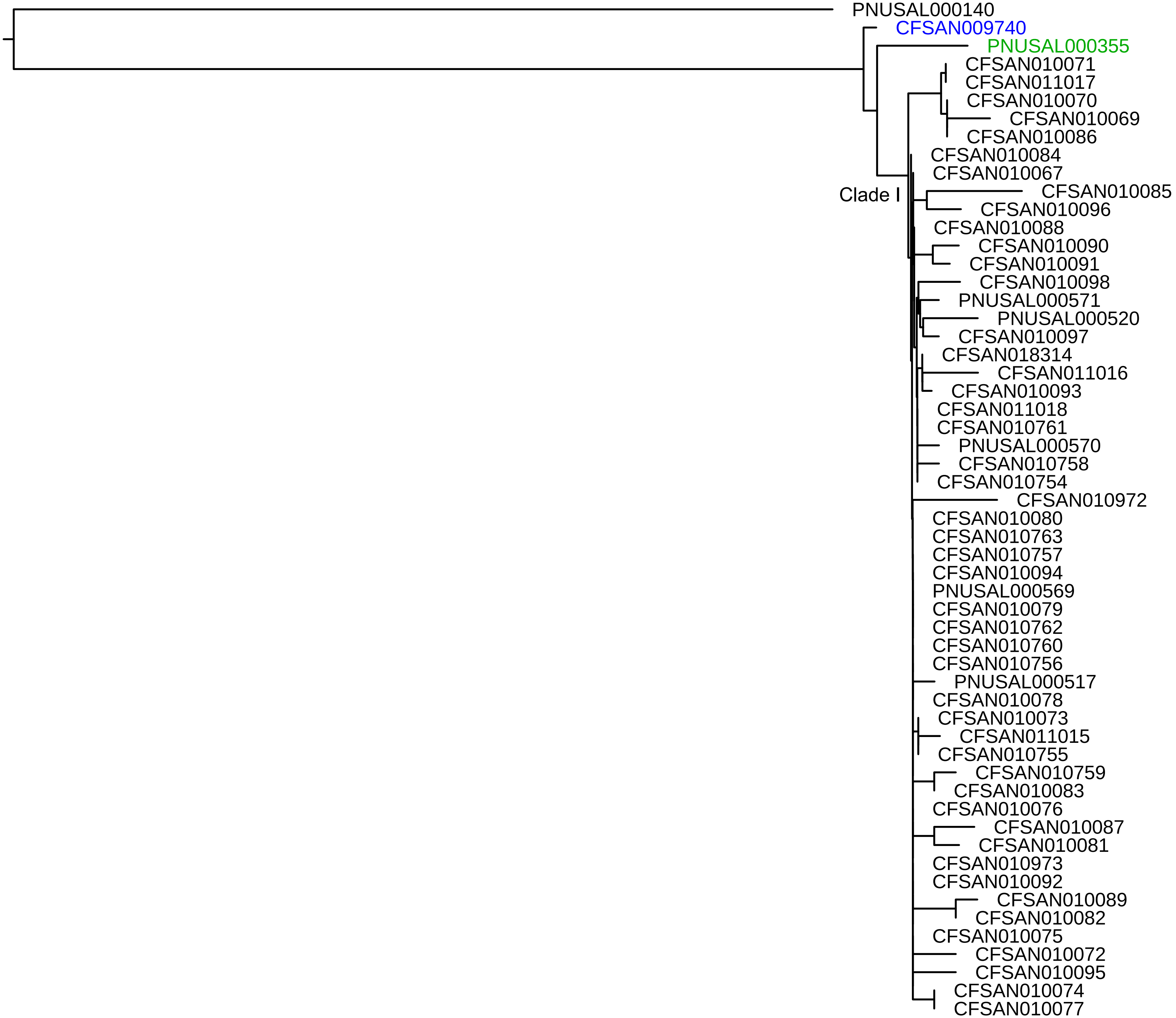

Figure S4. NJ phylogeny based on summary calls of cgMLST. The California and New York isolates are marked in green and blue color, respectively.

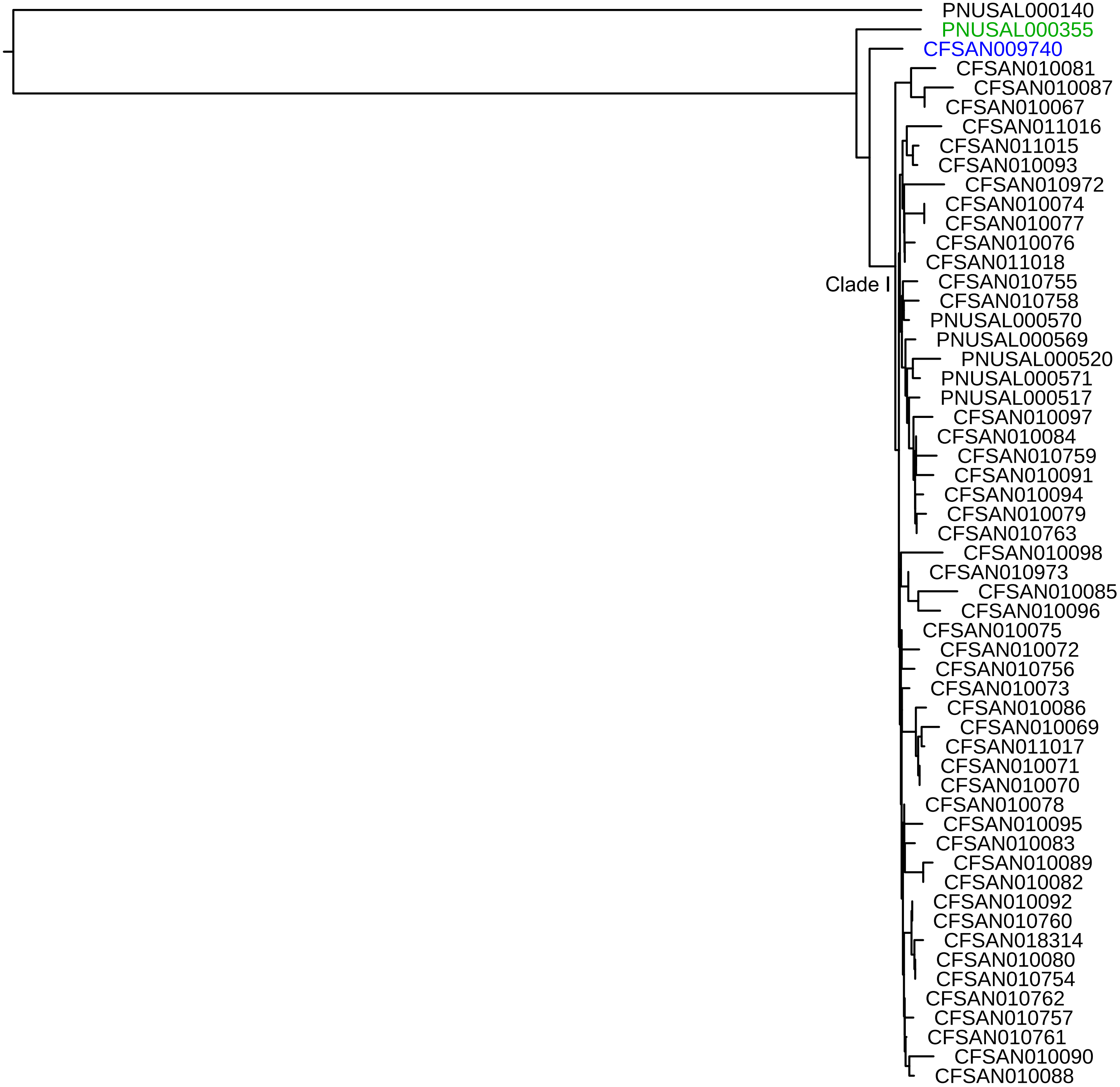

Figure S5. NJ phylogeny generated using assembly-based alleles of wgMLST. The California and New York isolates are marked in green and blue color, respectively.

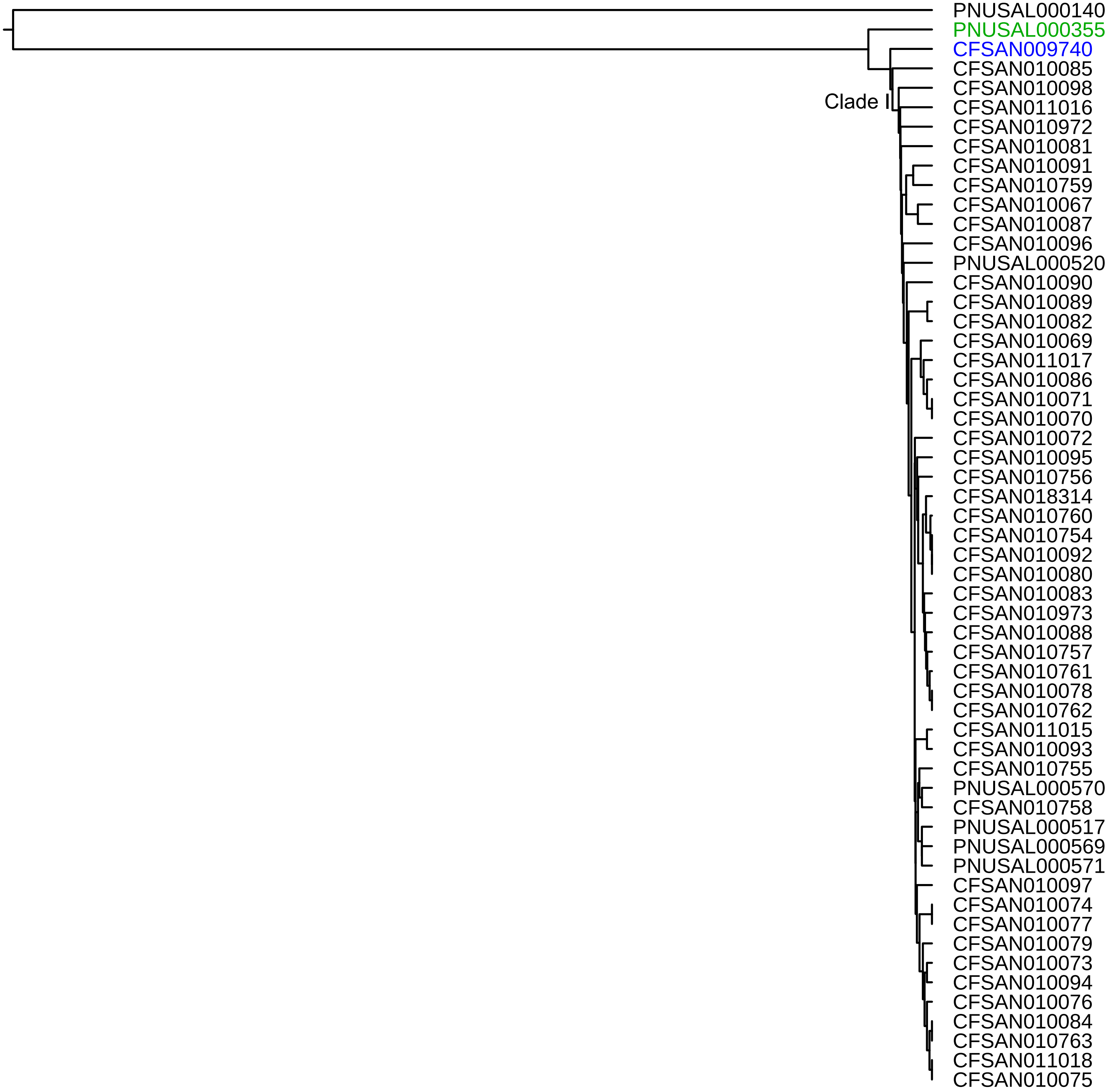

Figure S6. UPGMA phylogeny generated using assembly-based alleles of wgMLST. The California and New York isolates are marked in green and blue color, respectively.

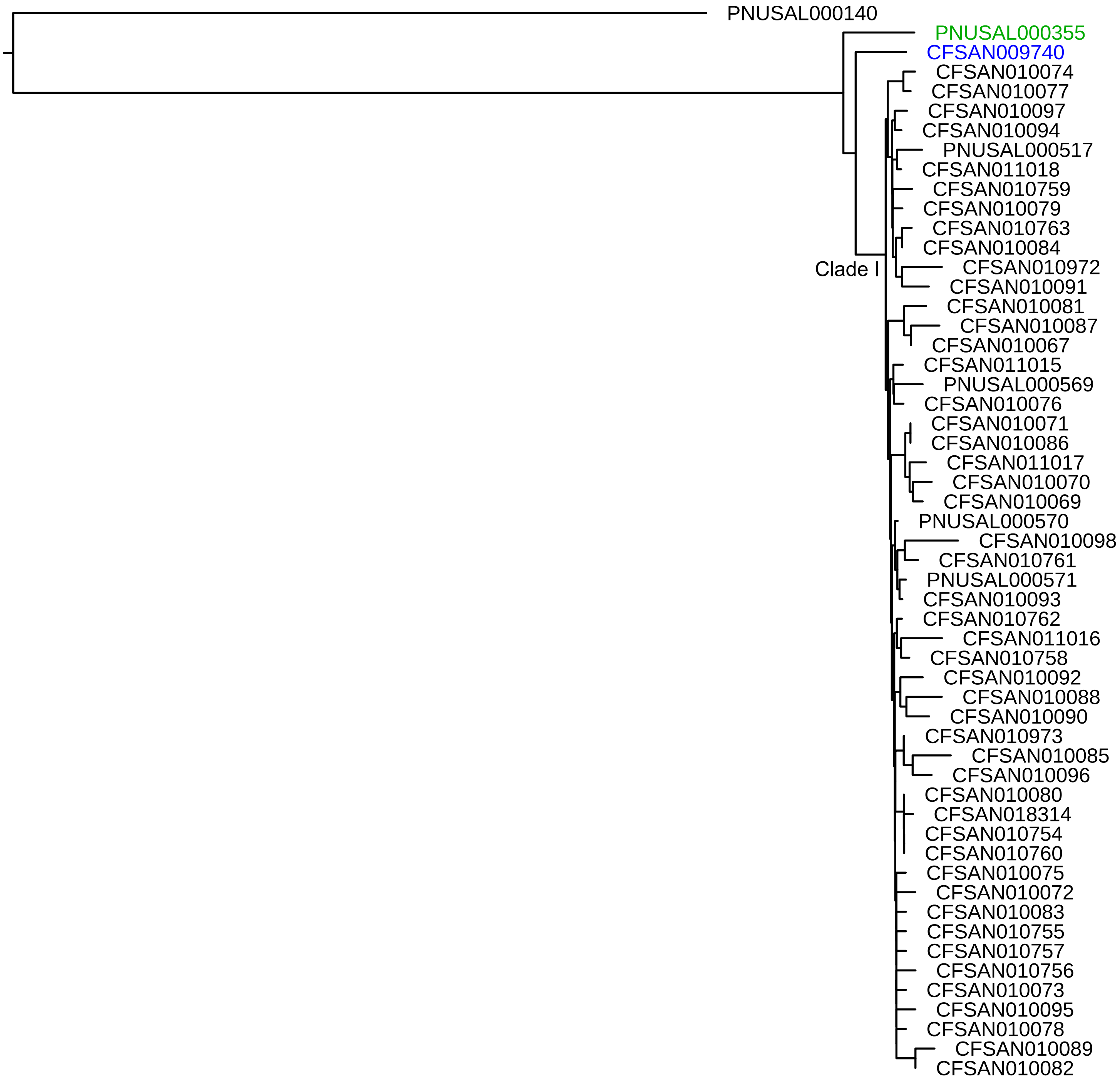

Figure S7. NJ phylogeny generated using assembly-free alleles of wgMLST. The California and New York isolates are marked in green and blue color, respectively.

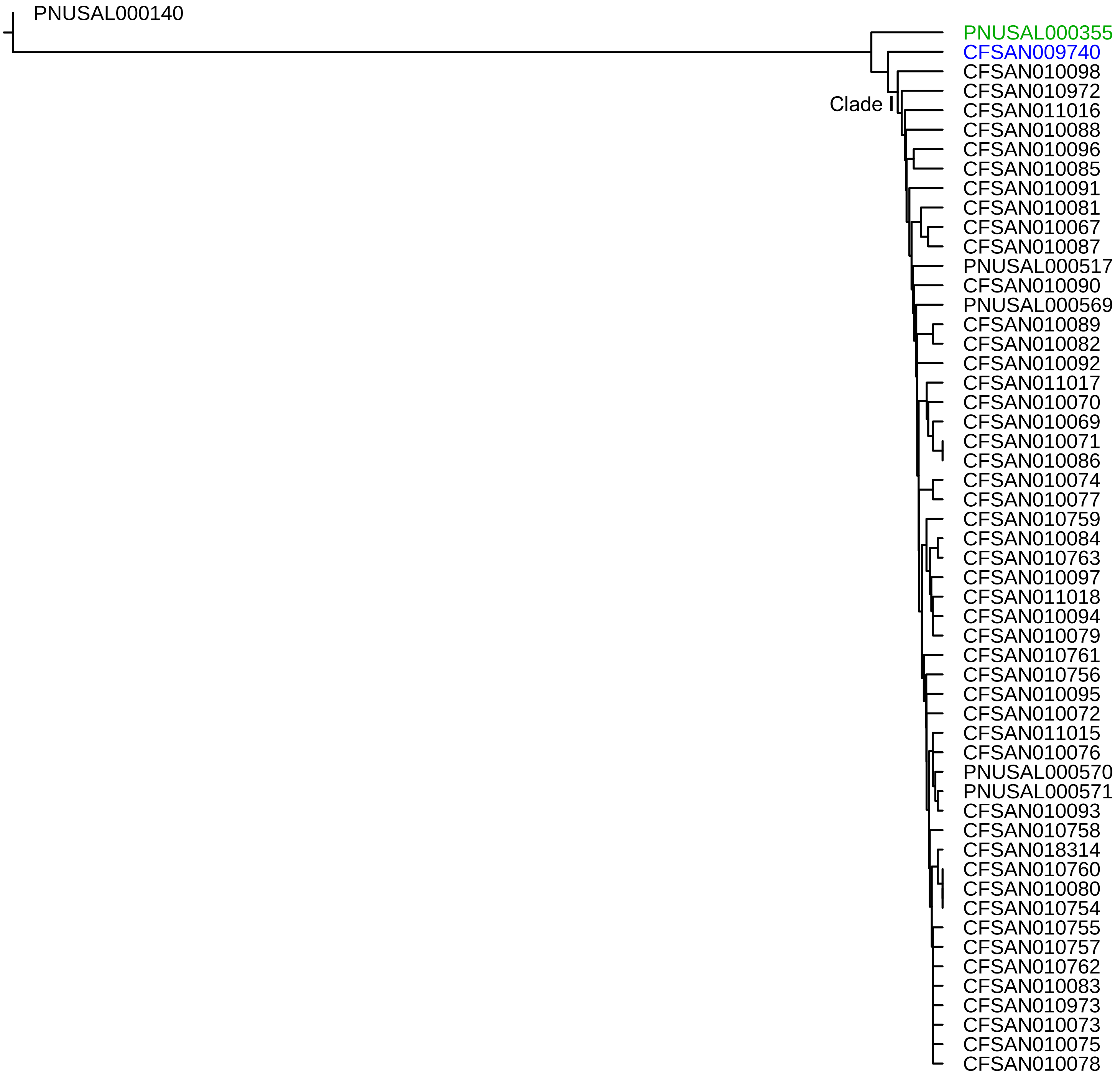

Figure S8. UPGMA phylogeny generated using assembly-free alleles of wgMLST. The California and New York isolates are marked in green and blue color, respectively.

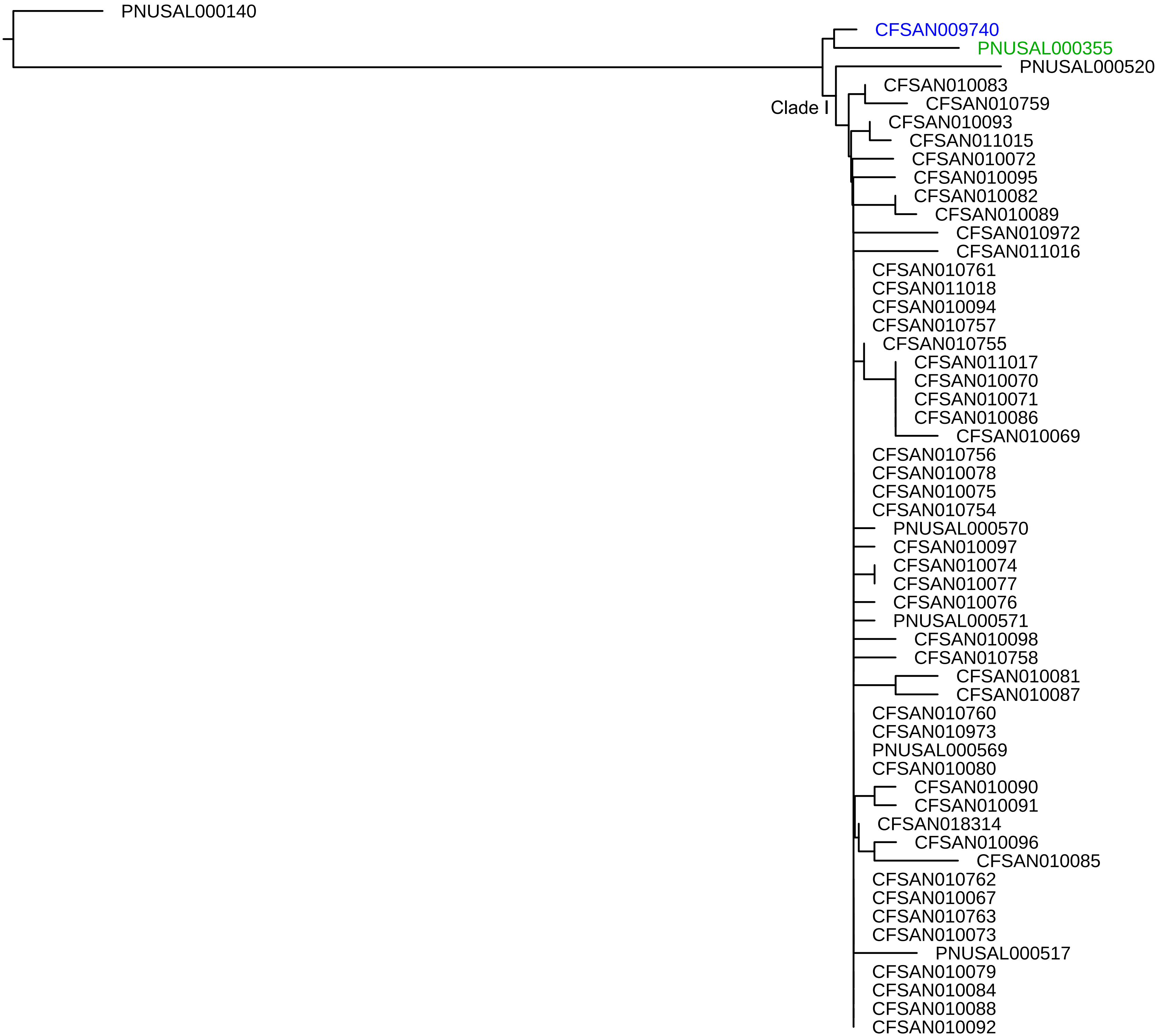

Figure S9. NJ phylogeny generated using assembly-based allele calls of cgMLST. The California and New York isolates are marked in green and blue color, respectively.

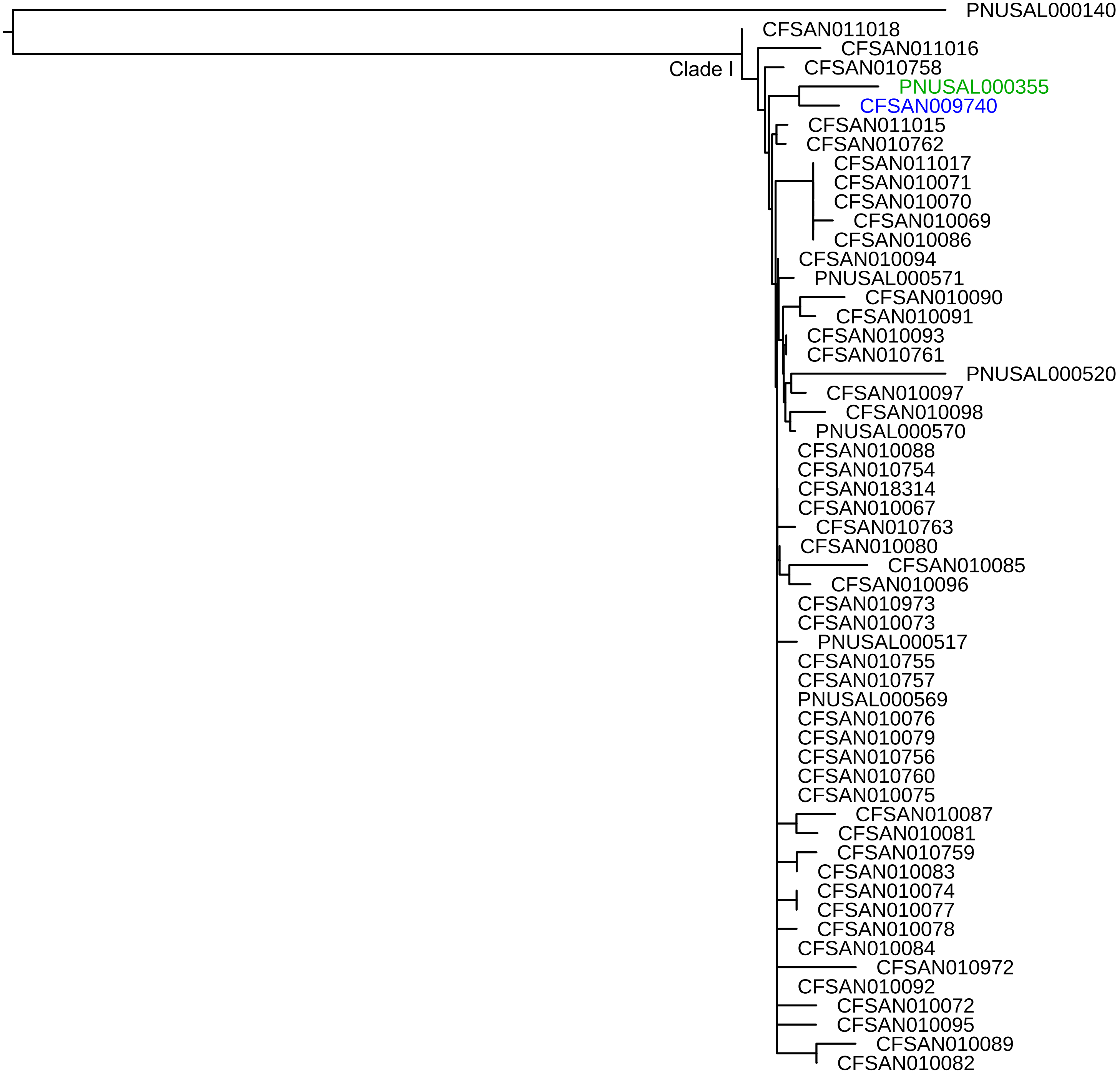

Figure S10. NJ phylogeny generated using assembly-free allele calls of cgMLST. The California and New York isolates are marked in green and blue color, respectively.

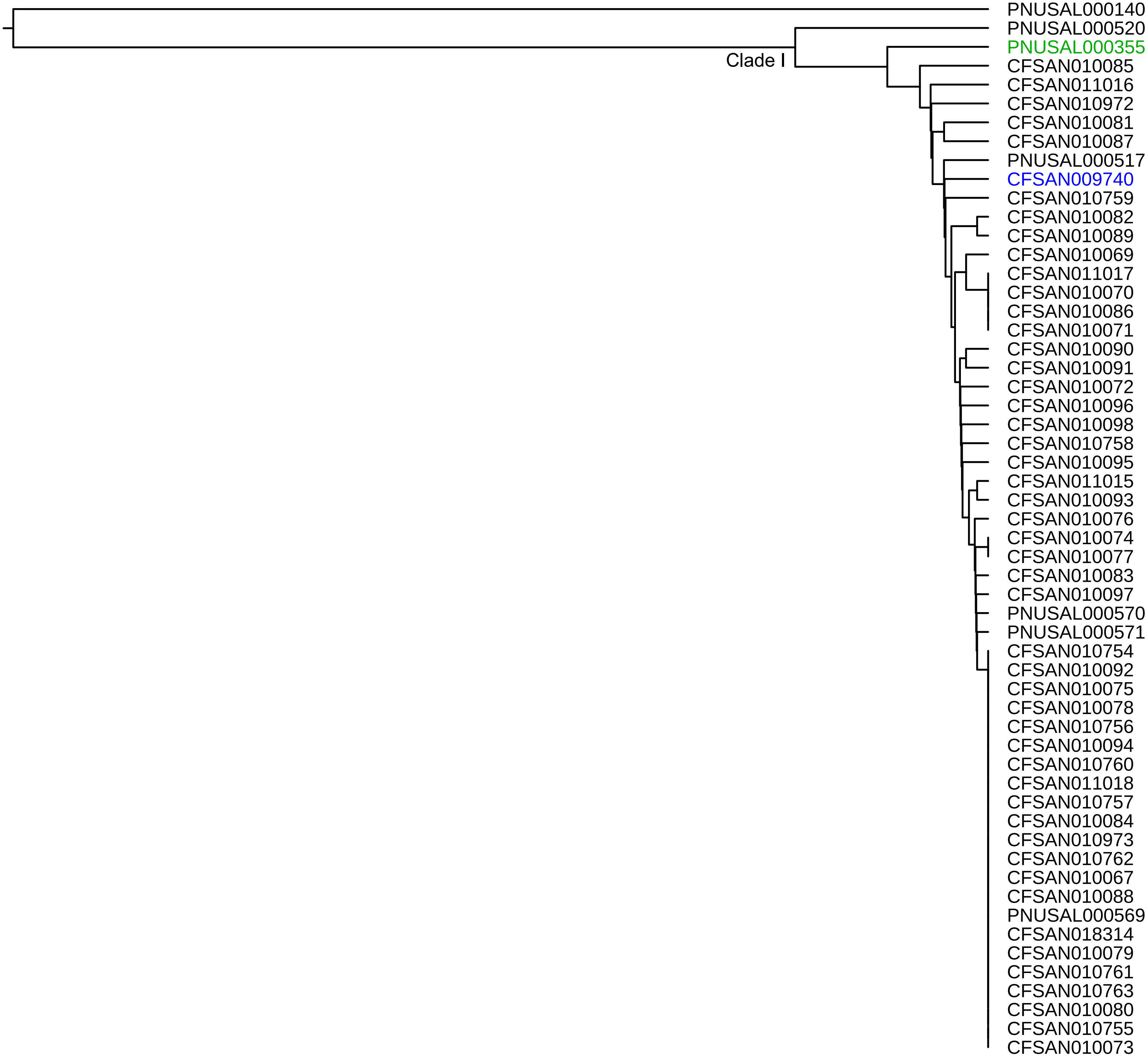

Figure S11. UPGMA phylogeny generated using assembly-based allele calls of cgMLST. The California and New York isolates are marked in green and blue color, respectively.

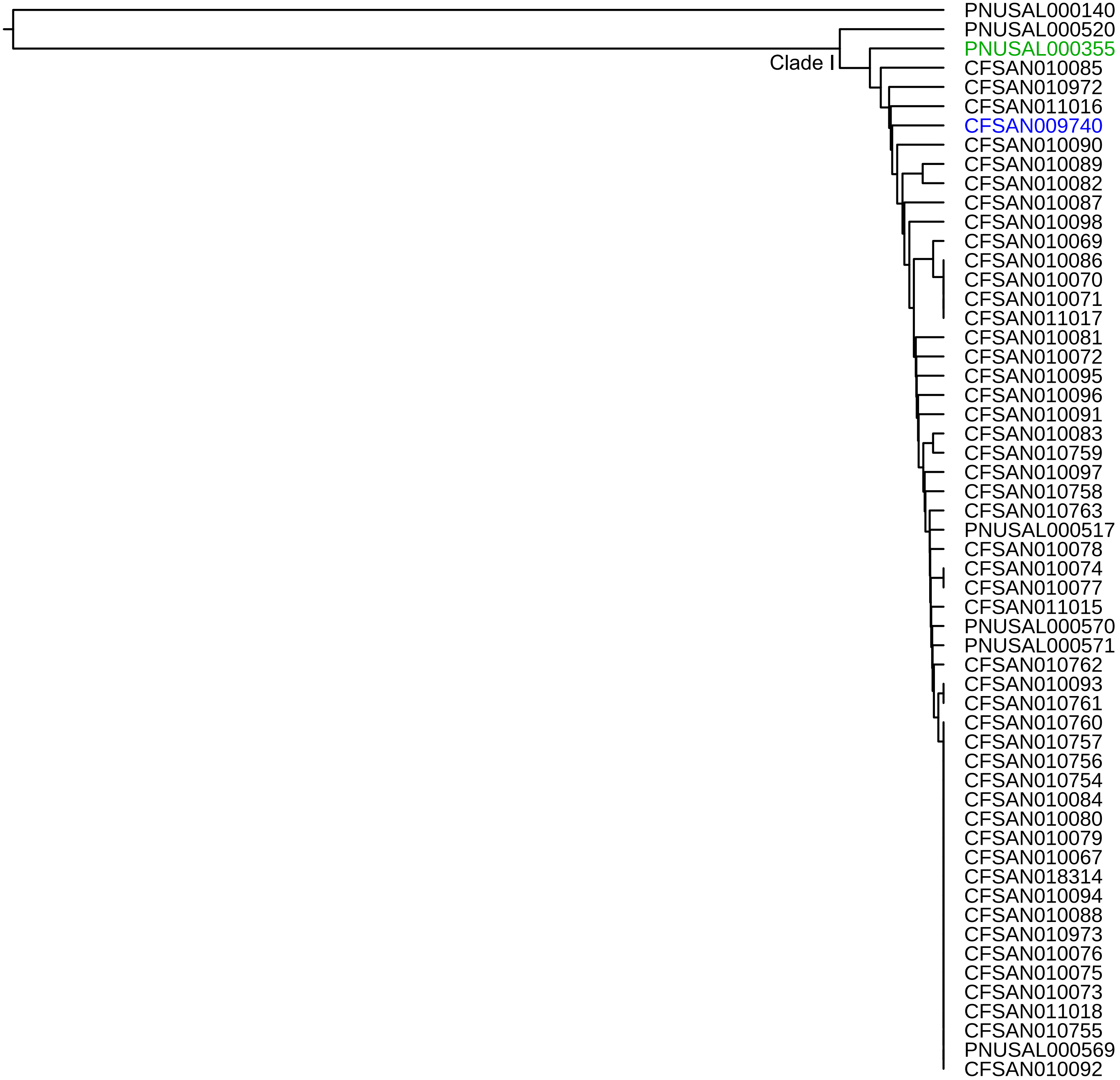

Figure S12. UPGMA phylogeny generated using assembly-free allele calls of cgMLST. The California and New York isolates are marked in green and blue color, respectively.
